# Frizzled‐9 Expression Is Associated With Aggressive Clinicopathological Features and Reduced Overall Survival in Invasive Breast Carcinoma

**DOI:** 10.1111/pin.70104

**Published:** 2026-03-10

**Authors:** Daniel Rodrigues de Bastos, Ricardo Cesar Cintra, Adhemar Longatto‐Filho, Lara Termini

**Affiliations:** ^1^ Center for Translational Research in Oncology (LIM/24), Instituto do Cancer do Estado de Sao Paulo, Hospital das Clinicas HCFMUSP, Faculdade de Medicina Universidade de Sao Paulo São Paulo Brazil; ^2^ Facultad de Ciencias Médicas Universidad Privada del Este filial Ciudad del Este Paraguay; ^3^ Medical Laboratory of Medical Investigation (LIM/14), Department of Pathology São Paulo University Faculty of Medicine São Paulo Brazil; ^4^ Life and Health Sciences Research Institute (ICVS) Minho University Braga Portugal; ^5^ Teaching and Research Institute, Molecular Oncology Research Center Barretos Cancer Hospital—Pio XII Foundation São Paulo Brazil; ^6^ Comprehensive Center for Precision Oncology (C2PO) Universidade de Sao Paulo São Paulo Brazil

**Keywords:** breast cancer, FZD9, prognostic biomarker, tissue microarray, Wnt signaling pathway

## Abstract

The Wnt/Frizzled signaling pathway is implicated in tumor progression, yet the expression patterns and regulatory dynamics of Frizzled class receptor 9 (FZD9) in breast cancer remain poorly defined. This study evaluated FZD9 protein expression in breast tumors and explored its transcriptional modulation in breast cancer cell lines exposed to cytotoxic and epigenetic agents. Immunohistochemical analysis was performed in 81 breast cancer cases representing major molecular subtypes. In parallel, breast cancer cell lines were treated with trichostatin A, 5‐aza‐2′‐deoxycytidine, cisplatin, doxorubicin, paclitaxel, and ionizing radiation, followed by quantification of FZD9 mRNA levels by RT‐qPCR. FZD9 protein expression was more frequent in HER2‐enriched tumors, cases with high Ki‐67 index, and advanced T‐stage. FZD9‐positive tumors were associated with reduced overall survival, whereas relapse‐free survival showed no significant difference. Baseline FZD9 transcript levels varied substantially across cell lines, and transcriptional responses to chemotherapy, radiation, and epigenetic treatment were highly context‐dependent, with divergent patterns observed according to molecular background. Collectively, these findings indicate that FZD9 expression in breast cancer is heterogeneous, associated with aggressive clinicopathological features, and dynamically modulated by therapeutic exposures, supporting its consideration as an exploratory marker of tumor aggressiveness and therapy‐related biological responses.

## Graphical abstract

Graphical summary illustrating the evaluation of FZD9 in breast cancer. FZD9 protein expression was assessed by immunohistochemistry in 81 breast tumor samples and further investigated in breast cancer cell lines exposed to cytotoxic and epigenetic treatments. Higher FZD9 expression was associated with aggressive clinicopathological features and poorer overall survival. Experimental assays demonstrated heterogeneous transcriptional responses to therapeutic agents, underscoring the context‐dependent role of FZD9 in breast cancer biology. FZD9, Frizzled‐9; Wnt, Wingless‐related integration site; IHC, immunohistochemistry; TSA, trichostatin A; 5AZA, 5‐aza‐2’‐deoxycytidine.

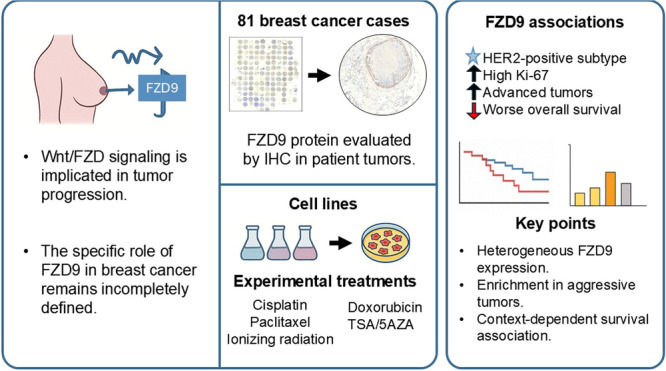

## 1 Introduction

Breast cancer (BC) remains the most diagnosed malignant tumor among women worldwide, with ~2.3 million new cases and around 670,000 deaths in 2022, accounting for 11.6% of global cancer incidence and 6.9% of cancer‐related mortality [1]. In Brazil, the National Cancer Institute (INCA) estimates an average of 73,610 new BC cases per year for the 2023–2025 period, with an age‐adjusted risk rate of 66.5 per 100,000 women in 2024, confirming BC as the most frequent cancer type in the country. Furthermore, breast cancer accounts for 16.1% of all cancer‐related deaths among Brazilian women, ranking as the leading cause of cancer mortality in this population [2]. Despite therapeutic advances, the biological heterogeneity of BC contributes to distinct clinical outcomes, with patients facing high risks of recurrence and treatment resistance [3, 4].

The clinicopathological classification of breast cancer has enabled the subdivision of the disease into subgroups with distinct prognostic and therapeutic profiles, based on the immunohistochemical assessment of hormone receptors (estrogen and progesterone), human epidermal growth factor receptor 2 (HER2), and the proliferative index Ki‐67. In clinical practice, these immunohistochemical biomarkers are widely used as surrogate classifiers, allowing the categorization of tumors into groups such as Luminal A, Luminal B, HER2‐positive, and triple‐negative breast cancer (TNBC) [5, 6, 7]. It is important to note, however, that intrinsic molecular subtypes defined by gene expression profiling (e.g., basal‐like and luminal A) are not fully equivalent to surrogate classifications based on immunohistochemistry, such as triple‐negative or luminal‐like tumors, although substantial overlap exists between these categories.

Among the molecular pathways implicated in breast carcinogenesis, the Wnt signaling pathway stands out due to its aberrant activation, which has been associated with cancer stem cell maintenance, metastasis, and therapeutic resistance [8, 9]. The Frizzled (FZD) receptors, which comprise a subfamily of seven‐transmembrane domain proteins, are the main mediators of Wnt signal transduction and perform highly context‐dependent functions, acting as either promoters or inhibitors of tumor growth depending on the cellular and molecular environment [10].

Among the members of this family, the Frizzled class receptor 9 (FZD9) has attracted increasing attention. Located on chromosome 7q11.23, FZD9 is encoded by a single exon and translated into a 591‐amino‐acid protein expressed in tissues such as the brain, eyes, kidneys, and skeletal muscle [11]. Its involvement has been reported in several solid tumors, including pancreatic, lung, cervical, bone (osteosarcoma), and brain (astrocytoma) cancers, displaying heterogeneous expression patterns and diverse biological functions, such as regulation of apoptosis, ossification, and intracellular calcium control [9, 12, 13, 14, 15, 16, 17].

In the context of breast cancer, available data remains limited and is restricted to a few published studies. One of the most relevant investigations was conducted by Hachim et al. (2020), who analyzed FZD9 protein expression by immunohistochemistry (IHC) in a breast cancer patient cohort and demonstrated significantly higher expression in TNBC tumors compared to other molecular subtypes [18]. Similarly, our group conducted a comprehensive analysis using public transcriptomic datasets and identified FZD9 as a differentially expressed gene in basal‐like/TNBC breast tumors, with significantly elevated levels in these subtypes. Moreover, our findings showed that higher FZD9 expression was associated with worse overall survival, shorter relapse‐free survival, TP53 mutations, chemotherapy resistance, and reduced promoter methylation [19].

Building upon previous transcriptomic analyses that identified FZD9 as a gene associated with aggressive breast cancer phenotypes, particularly within basal‐like and triple‐negative tumors, the present study was designed as an exploratory investigation focusing on protein‐level expression and contextual regulation of FZD9 in breast cancer. Given the well‐recognized discordance that may occur between mRNA abundance and protein expression, as well as the influence of tumor heterogeneity and microenvironmental factors, we sought to evaluate FZD9 expression by immunohistochemistry in a tissue microarray cohort and to examine its transcriptional modulation in breast cancer cell lines exposed to cytotoxic and epigenetic agents. Rather than aiming to establish FZD9 as a definitive prognostic biomarker, this study intends to provide insight into its clinicopathological associations and context‐dependent biological behavior, thereby generating hypotheses for future validation in larger and independent cohorts.

## 2 Methodology

### 2.1 Ethical Approval and Study Population

This study was approved by the Research Ethics Committee of the Hospital das Clínicas, Faculty of Medicine, University of São Paulo (HCFMUSP; protocol no. 4.995.205). We retrospectively reviewed the medical records of 92 consecutive patients diagnosed with breast cancer. Clinicopathological data at the time of diagnosis, including histological malignancy grade, maximum tumor diameter, lymph node involvement, estrogen and progesterone receptor status, HER2 expression, and the immunohistochemical profile, were extracted from patient medical records. As this was a retrospective analysis, not all clinicopathological variables were available for every case. Given the limited number of cases and the availability of tumor tissue, cases were retained in the study provided that adequate biopsy material was available for FZD9 immunohistochemical evaluation.

### 2.2 Tissue Microarray Processing and Immunostaining

Formalin‐fixed, paraffin‐embedded breast carcinoma specimens were arrayed into a tissue microarray (TMA) and sectioned at 4 µm. Sections were deparaffinized, rehydrated, and subjected to antigen retrieval before automated staining on a Ventana BenchMark GX platform (Roche Diagnostics, Basel, Switzerland). FZD9 was detected with rabbit polyclonal anti‐FZD9 (ThermoFisher Scientific, Waltham, MA, USA, PA5‐27119; 1:500), followed by visualization with the UltraView Universal DAB Detection Kit (Roche Diagnostics, Basel, Switzerland) according to the manufacturer's instructions. Each staining run included both positive and negative controls to verify specificity and consistency.

All TMA cores were independently evaluated by a board‐certified pathologist with expertise in breast pathology, who was blinded to all clinical and outcome data. Staining intensity (0–3) and extent (0–3) were recorded for each of three replicate cores per tumor; the mean intensity and extent were multiplied to yield an H‐score (H = mean intensity × mean extent). For exploration analyses, H‐scores were analyzed as continuous variables. For subgroup comparisons and survival analyses, FZD9 expression was dichotomized into positive and negative groups using the statistically optimized cut‐point determined by the *surv_cutpoint* function (survminer R package) [20]. To further support antibody specificity and staining reproducibility, immunohistochemical standardization experiments were performed using breast cancer and glioblastoma tissue sections, including serial antibody dilutions and parallel negative controls processed without primary antibody (Supporting Figures 1 and 2).

### 2.3 Cell Line Culture and Treatment

Breast carcinoma cell lines Hs578T (HTB‐126), MDA‐MB‐231 (HTB‐26), SK‐BR‐3 (HTB‐30), HCC70 (CRL‐2315), MCF7 (HTB‐22), BT‐474 (HTB‐20) and BT‐549 (HTB‐122) were all obtained from the American Type Culture Collection (ATCC, Manassas, VA, USA) and cultured in DMEM or RPMI 1640 (Gibco, ThermoFisher Scientific, Waltham, MA, USA), as recommended by ATCC, each medium supplemented with 10% fetal bovine serum and 1% penicillin–streptomycin (Gibco, ThermoFisher Scientific, Waltham, MA, USA). All lines were maintained at 37°C in a humidified 5% CO₂ atmosphere, with medium renewal every 48 h. Cells were passaged at 50%–60% confluence by treatment with 0.25% trypsin–EDTA (Gibco, ThermoFisher Scientific, Waltham, MA, USA) and reseeded at appropriate densities. Mycoplasma contamination was routinely excluded before and after all experimental procedures using validated detection protocols.

### 2.4 Treatment Conditions and Dosimetry

Epigenetic modulators 5‐aza‐2′‐deoxycytidine (5‐AZA; Sigma‐Aldrich, St. Louis, MO, USA) and Trichostatin‐A (TSA; Cayman Chemical, Ann Arbor, MI, USA) were titrated by Alamar Blue viability assay (Thermo Fisher Scientific, Waltham, MA, USA) to define the highest non‐cytotoxic concentrations, i.e., doses that did not alter cell morphology or proliferation. Chemotherapeutics cisplatin (Sigma‐Aldrich, St. Louis, MO, USA), doxorubicin (Pfizer, New York, NY, USA) and paclitaxel (Bristol‐Myers Squibb, Princeton, NJ, USA) were applied at ½IC₅₀ values obtained from the CancerRxGene database [21], with SK‐BR‐3 concentrations adapted from Hai et al. due to unavailable public IC₅₀ data. Ionizing radiation (6 Gy single fraction) was delivered using an RS2000 Biological Irradiator (Rad Source Technologies, Suwanee, GA, USA) with dose uniformity confirmed by Gafchromic film dosimetry. For epigenetic experiments, cells were maintained under continuous exposure to 5‐AZA or TSA for 7 days, with daily medium replacement containing fresh compound.

### 2.5 Gene Expression Analysis by RT‐qPCR

After treatment, 2 µg of total RNA was reverse transcribed using the High‐Capacity cDNA Reverse Transcription Kit (Thermo Fisher Scientific, Waltham, MA, USA). Quantitative PCR was then performed using SYBR Green chemistry on the 7500 Real‐Time PCR System (Applied Biosystems, Thermo Fisher Scientific, Foster City, CA, USA). Amplification was carried out using specific oligonucleotide primers targeting FZD9 (RefSeq NM_003508.3) and the endogenous control GAPDH (RefSeq NR_152150.2), with the following sequences (5′–3′): FZD9 forward: GTGGAGATCCCCATGTGCC and FZD9 reverse: CCGAAGTTGAACTGCTCCAT; GAPDH forward: GACTGTGGTCATGAGTCCTCCC; and GAPDH reverse: CAAGATCATCAGCAATGCCTCC (synthesized by Thermo Fisher Scientific, Waltham, MA, USA). Relative FZD9 expression was normalized to GAPDH and calculated using the 2^^−ΔΔCT^ method. All reactions were performed in technical triplicate.

### 2.6 Statistical Analysis

Statistical analyses were performed using GraphPad Prism version 6.0 (GraphPad Software, San Diego, USA) and R software (version 4.2.1). Associations between FZD9 expression and categorical clinicopathological variables were evaluated using the chi‐square test or, when appropriate, Fisher's exact test. Comparisons between groups for continuous variables (such as FZD9 IHC score) were performed using the Mann–Whitney U test. For survival analysis, Kaplan–Meier curves were generated for overall survival (OS) and relapse‐free survival (RFS), with differences between groups assessed using the log‐rank test. The cut‐off value for categorizing FZD9 expression (positive vs. negative) was determined automatically using the surv_cutpoint function from the survminer R package [20], which identifies statistically relevant cut‐off points for survival analysis. In addition, multivariable survival analysis was conducted using Cox proportional hazards regression to estimate adjusted hazard ratios (HRs) and 95% confidence intervals (CIs), including ER, Ki‐67 category, TNM stage (I/II vs. III/IV), and FZD9 expression as covariates; cases with missing values for any included covariate were excluded from the multivariable model. A *p*‐value < 0.05 was considered statistically significant for all analyses.

## 3 Results

### 3.1 Immunohistochemical Profiling of FZD9 and Its Clinicopathological Correlations in Breast Cancer

**Figure 1:**
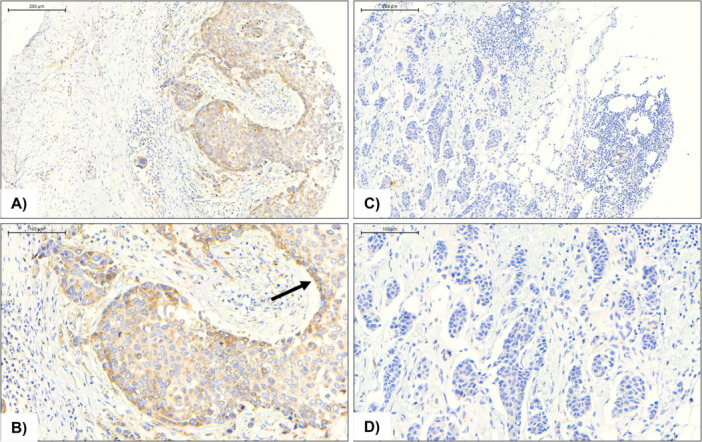
Representative immunohistochemical staining for FZD9 in breast cancer samples from a tissue microarray. Panels illustrate varying intensities of FZD9 expression: positive (A, B; note the arrow in B pointing to the most prominent staining at the tumor border), and negative (C, D) patterns. Images correspond to TMA cores evaluated at 10× (A–C) and 20× (B–D) magnification.

FZD9 protein expression was examined by immunohistochemistry in a tissue microarray composed of 92 breast tumor samples. Of these, 11 cases were excluded from analysis, seven due to incomplete clinicopathological information that precluded proper tumor classification, and four due to sample loss during the immunostaining process. The final cohort included 81 evaluable cases, each represented in triplicate to ensure consistency and reliability of interpretation. Representative TMA cores revealed heterogeneous staining patterns, with examples of positive (Figure 1A, B) and negative (Figure 1C, D) FZD9 staining, illustrating the variable distribution of this marker across the breast cancer cohort.

Among 81 evaluable invasive breast carcinoma specimens, FZD9 expression showed no significant association with patient age (*p* = 0.6964), with most tumors in both FZD9‐positive and FZD9‐negative groups arising in women over 50 years. Likewise, estrogen receptor status (*p* = 0.4595) and progesterone receptor status (*p* = 0.5664) did not differ significantly between groups, although ER and PR positivity were marginally more frequent in FZD9‐negative tumors (76.4% and 69.1%, respectively) than in FZD9‐positive tumors (68.2% and 62.5%).

By contrast, HER2 positivity was significantly enriched in FZD9‐positive cases (33.3% *vs.* 9.4%; *p* = 0.0184), although this observation was based on a limited number of HER2‐positive tumors. High Ki‐67 expression was more common in FZD9‐positive tumors (75.0% *vs.* 63.2%), but this difference did not reach statistical significance (*p* = 0.3019). Histological subtype distribution was similar across groups (*p* = 0.7821), with invasive ductal carcinoma predominating.

Although overall TNM stage distribution did not differ significantly (*p* = 0.4288), advanced stage (III–IV) was more prevalent among FZD9‐positive tumors. Tumor size correlated positively with FZD9 expression: 91.7% of FZD9‐positive tumors were stage II–IV compared with 70.2% of FZD9‐negative tumors (*p* = 0.0371). Molecular subtype analysis also revealed a significant association (*p* = 0.0351), with HER2‐enriched tumors overrepresented in the FZD9‐positive cohort (25.0%), while luminal A and triple‐negative subtypes predominated in FZD9‐negative cases (Table 1).

**Table 1:** Clinicopathological parameters according to FZD9 immunohistochemical expression in breast cancer.

Clinical parameters	Negative		Positive		*p*‐value
	*n*	%	*n*	%	
**Age**					0.6964
≤ 50	12	21.10	6	25.00	
> 50	45	78.90	18	75.00	
**ER**					0.4595
Negative	13	23.60	7	31.80	
Positive	42	76.40	15	68.20	
**PR**					0.5664
Negative	17	30.90	9	37.50	
Positive	38	69.10	15	62.50	
**HER2**					0.0184
Negativo	48	90.60	16	66.70	
Positivo	5	9.40	8	33.30	
**Ki67**					0.3019
Negative	21	36.80	6	25.00	
Positive	36	63.20	18	75.00	
**Histological diagnosis**					0.7821
IDC	50	90.90	24	100.00	
Metaplastic	3	5.50	0	0.00	
Mixed ductal and lobular	1	1.80	0	0.00	
Mixed ductal and micropapillary	1	1.80	0	0.00	
**TNM classification**					0.4288
I	13	22.80	2	8.30	
II	25	43.90	12	50.00	
III	15	26.30	7	29.20	
IV	4	7.00	3	12.50	
**Tumor size**					0.0371
I	17	29.80	2	8.30	
II/III/IV	40	70.20	22	91.70	
**Molecular classification**					0.0351
Luminal A	13	23.20	3	12.50	
Luminal B	29	51.80	12	50.00	
HER2	2	3.60	6	25.00	
TNBC	12	21.40	3	12.50	

Abbreviations: ER, estrogen receptor; HER2, human epidermal growth factor receptor 2; IDC, invasive ductal carcinoma; Ki‐67, cellular proliferation marker Ki‐67; PR, progesterone receptor; TNBC, triple‐negative breast cancer; TNM, tumor, node, metastasis classification system.

### 3.2 FZD9 Expression Score and Its Relationship With Tumor Characteristics

**Figure 2:**
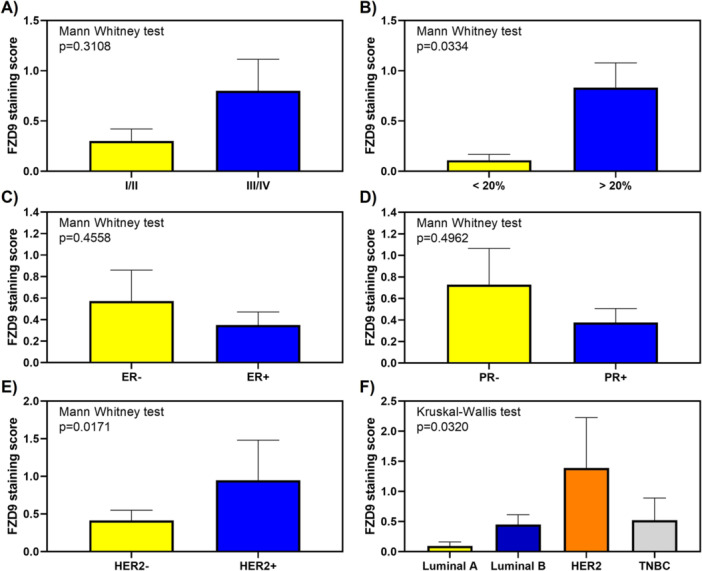
Distribution of FZD9 immunohistochemical expression score across clinicopathological variables in invasive breast carcinoma. Boxplots represent the variation of the FZD9 IHC score (product of staining intensity and extent) according to: (A) TNM clinical stage; (B) Ki‐67 status (cut‐off: 20%); (C) estrogen receptor (ER) status; (D) progesterone receptor (PR) status; (E) HER2 status; and (F) molecular subtype. Differences in score distribution were assessed between categories to explore expression profiles across tumor subgroups.

The distribution of FZD9 H‐scores did not differ significantly across TNM stages I/II–III/IV (Figure 2A). In contrast, tumors with a high proliferative index (Ki‐67 ≥ 20%) exhibited significantly higher median H‐scores than those with low proliferative activity (Ki‐67 < 20%; Figure 2B).

Estrogen receptor–negative tumors had higher median H‐scores than ER‐positive cases (Figure 2C), and progesterone receptor–negative tumors likewise showed elevated H‐scores relative to PR‐positive tumors (Figure 2D). HER2‐positive tumors demonstrated markedly higher H‐scores compared with HER2‐negative tumors (Figure 2E). Finally, by molecular subtype, HER2‐enriched tumors showed the highest median H‐scores, while luminal B and triple‐negative tumors exhibited comparable and overlapping expression patterns, and luminal A tumors consistently showed lower expression (Figure 2F).

### 3.3 Prognostic Impact of FZD9 Expression on Patient Survival

**Figure 3:**
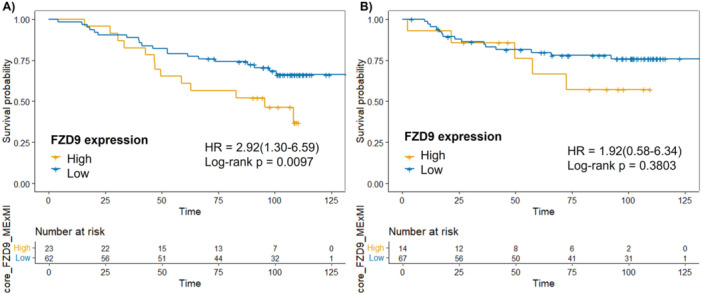
Kaplan–Meier survival curves according to FZD9 staining status in patients with invasive breast carcinoma. FZD9 expression was dichotomized as positive or negative based on optimal cut‐off values determined using the survminer R package. (A) Overall survival. (B) Relapse‐free survival. Number at risk is displayed below each curve.

Kaplan–Meier analysis demonstrated that patients with FZD9‐positive tumors had significantly reduced overall survival compared with those with FZD9‐negative tumors (Figure 3A; *p* = 0.0097; hazard ratio (HR) = 2.92; 95% confidence interval (CI) = 1.30–6.59). In contrast, relapse‐free survival did not differ significantly between the two groups (Figure 3B; *p* = 0.3803; HR = 1.92; 95% CI = 0.58–6.34), with overlapping survival curves and a limited number of relapse events.

To contextualize FZD9's prognostic value, we assessed conventional clinicopathological markers (Supporting Figure 3). Estrogen receptor–positive and progesterone receptor–positive status was both associated with improved overall survival (*p* = 0.00149 for ER; *p* = 0.00149 for PR). HER2 status did not significantly affect survival (*p* = 0.256). High proliferative index (Ki‐67 ≥ 20%) predicted poorer overall survival (*p* = 0.000327), and patients with advanced stage (III/IV) showed significantly worse outcomes than those with early‐stage disease (*p* = 0.000837). To further assess the independent prognostic value of FZD9, a multivariable Cox proportional hazards regression analysis was performed (Table 2). After adjustment for estrogen receptor status, Ki‐67 category, and TNM stage, FZD9 positivity remained independently associated with reduced overall survival (HR = 2.65; 95% CI = 1.13–6.23; *p* = 0.024). Advanced TNM stage (III/IV) was also an independent predictor of poor outcome (HR = 3.31; 95% CI = 1.24–8.83; *p* = 0.017), whereas estrogen receptor status and Ki‐67 did not retain independent prognostic significance in the adjusted model.

**Table 2:** Multivariable Cox proportional hazards regression analysis for overall survival in breast cancer patients.

Variable	HR (Exp(B))	95% CI (Lower–Upper)	*p* value
ER (positive vs. negative)	0.92	0.37–2.31	0.852
Ki‐67 (≥ 20% vs. < 20%)	2.85	0.76–10.72	0.119
TNM stage (III/IV vs. I/II)	3.31	1.24–8.83	0.017
FZD9 (positive vs. negative)	2.65	1.13–6.23	0.024

### 3.4 FZD9 Baseline and Epigenetic Modulator–Induced Expression

**Figure 4:**
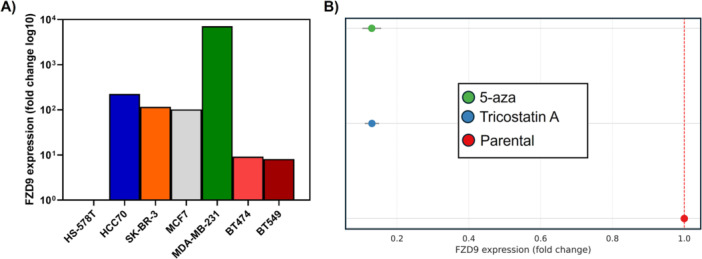
FZD9 baseline expression and response to epigenetic treatment in breast cancer cell lines. (A) Relative expression of *FZD9* mRNA across seven breast cancer cell lines, measured by RT‐qPCR using HS‐578T as reference (fold change = 1). (B) *FZD9* expression in HS‐578T cells after treatment with 5‐aza‐2′‐deoxycytidine (5‐AZA) or Trichostatin‐A (TSA), represented as mean ± SD of biological triplicates.

FZD9 mRNA expression was quantified by RT‐qPCR in a panel of breast cancer cell lines. Marked inter‐line variability was observed, reflecting biological heterogeneity rather than absolute expression levels. MDA‐MB‐231 showed the highest expression, with a fold change of 7116.6 relative to HS‐578T, which was selected as a technical calibrator due to its consistently low baseline expression. HCC70, SK‐BR‐3, and MCF7 exhibited elevated levels, with fold changes of 223.7, 116.1, and 102.0, respectively. BT474 and BT549 presented intermediate expression, with fold changes of 9.2 and 8.2. HS‐578T exhibited the lowest expression and was used as calibrator for relative quantification **(**Figure 4A **)**.

In HS‐578T cells, treatment with 5‐aza‐2′‐deoxycytidine (5‐AZA) or Trichostatin‐A (TSA) was performed for 7 days, followed by evaluation of FZD9 transcript levels by RT‐qPCR. Both treatments resulted in decreased expression relative to untreated parental cells. Fold change values for TSA‐treated cells ranged from 0.11 to 0.15, and for 5‐AZA from 0.10 to 0.15, compared to a reference value of 1.0 in the parental condition (Figure 4B).

### 3.5 Transcriptional Response of FZD9 to Chemotherapy and Radiation in Breast Cancer Cells

**Figure 5:**
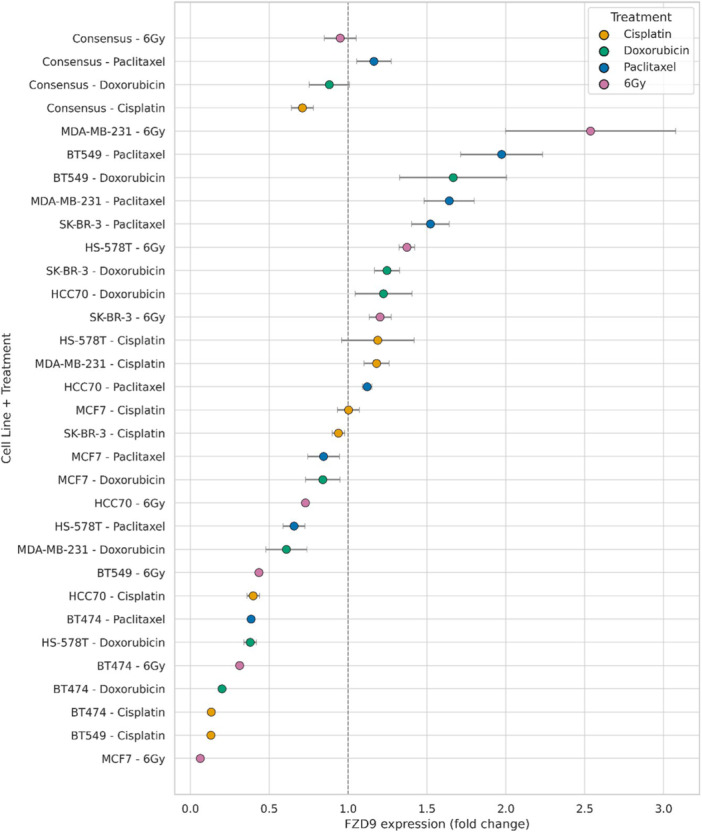
FZD9 fold change expression across breast cancer cell lines after exposure to cytotoxic agents. Relative FZD9 mRNA expression (fold change ±SD) was quantified by qPCR in seven breast cancer cell lines following treatment with cisplatin (yellow), doxorubicin (green), paclitaxel (blue), or 6 Gy ionizing radiation (purple). Data were normalized to the respective untreated controls. Color‐coded dots represent mean expression per condition; error bars indicate standard deviation. Consensus values per treatment are displayed at the bottom for comparison across conditions.

Quantitative analysis of FZD9 mRNA levels was performed across seven breast cancer cell lines exposed to cisplatin, doxorubicin, paclitaxel, or 6 Gy ionizing radiation. Fold change values, derived from qPCR data and normalized to untreated controls, are displayed with corresponding standard deviations (Figure 5).

The forest plot reveals substantial variability in FZD9 transcriptional response across treatments and molecular subtypes. The highest levels of induction were observed in MDA‐MB‐231 following irradiation (2.98 ± 0.18) and in BT549 upon paclitaxel exposure (2.06 ± 0.09). In contrast, strong downregulation was recorded in MCF7 treated with radiation (0.06 ± 0.01) and in BT474 under cisplatin (0.13  ±  0.01), both representing luminal phenotypes. When comparing average responses (labeled as “Consensus”), all treatments exhibited values near or below baseline (fold change ≈1), with minor deviations: paclitaxel (1.02 ± 0.26), doxorubicin (0.97 ± 0.20), cisplatin (0.88 ± 0.28), and radiation (0.80 ± 0.33).

## 4 Discussion

Despite substantial therapeutic progress in breast cancer management, a considerable proportion of patients continue to exhibit suboptimal responses, frequently experiencing disease recurrence that significantly worsens clinical outcomes and survival rates [3, 22]. In this context, the discovery of novel biomarkers capable of contributing to clinicopathological stratification, treatment response prediction, and prognostic assessment remains a central focus of translational oncology research. Based on this rationale, our group previously conducted a study aimed at identifying potential prognostic targets [19], and the present work represents a direct extension of that effort, focusing on the clinical validation and characterization of protein expression for the identified marker.

In this study, we investigated FZD9 expression patterns in breast cancer through integrated clinical and experimental analyses. Immunohistochemical evaluation revealed that FZD9 protein levels were heterogeneous among tumor samples and showed associations with clinicopathological variables, including molecular subtype, HER2 positivity, and Ki67 labeling index above 20%. High FZD9 expression was associated with worse overall survival and remained independently associated with poor outcome after adjustment for established prognostic factors in multivariable analysis, supporting its potential biological relevance. However, the absence of a statistically significant association with relapse‐free survival likely reflects the limited number of recurrence events and the restricted size of the study population. Given the complexity of Wnt/FZD signaling and the well‐recognized discordance that may exist between transcriptomic and proteomic analyses, careful interpretation of survival endpoints and subtype‐specific findings is warranted.

In this context, an apparent divergence between the present protein‐level findings and our previous transcriptomic analyses warrants careful consideration. In our earlier in silico study, subtype stratification was primarily based on intrinsic molecular classification using PAM50, in which elevated FZD9 mRNA expression was observed not only in basal‐like tumors but also in the HER2‐enriched molecular subtype, as evidenced in both The Cancer Genome Atlas (TCGA) and the Sweden Cancerome Analysis Network—Breast (SCAN‐B) cohort [19]. By contrast, the present study relies on immunohistochemistry‐based surrogate classification, in which HER2 status reflects protein overexpression rather than intrinsic molecular subtype assignment. Therefore, the higher frequency of FZD9 protein positivity observed among HER2‐positive tumors in the current cohort is not inconsistent with our previous findings, but rather aligns with transcriptomic patterns observed in HER2‐enriched tumors classified by Prediction Analysis of Microarray 50 (PAM50). The apparent lack of concordance with triple‐negative tumors in the present analysis likely reflects the limited overlap between TNBC defined by immunohistochemistry and the basal‐like molecular subtype, as well as the small number of cases analyzed. More broadly, discrepancies between mRNA abundance and protein expression are well documented in cancer biology and may arise from post‐transcriptional regulation, differences in protein stability, intracellular trafficking, or ligand‐dependent receptor internalization. Moreover, transcriptomic analyses typically rely on bulk tumor RNA, whereas immunohistochemistry provides spatially resolved, cell‐specific information that may capture distinct biological states within the tumor microenvironment. Importantly, the limited number of HER2‐positive and triple‐negative cases in the present study population constrains definitive subtype‐level comparisons and underscores the exploratory nature of these findings. Nevertheless, the observation that FZD9 protein expression is enriched in biologically aggressive tumor contexts, irrespective of the classification framework applied, supports a role for FZD9 in breast cancer aggressiveness and highlights the need for further validation in larger, subtype‐balanced cohorts.

In the IHC analysis, we observed that in many FZD9‐positive cases, staining was evident at the invasive tumor front. We hypothesize that the presence of this marker at the tumor edge (Figure 1B) may play an important role in maintaining proliferative activity, as this region typically exhibits higher metabolic activity and greater access to nutrients compared to cells located in the more central tumor regions [23]. Another noteworthy feature of the IHC analysis is the clear cytoplasmic staining pattern, which was also observable in the study by Hachim and colleagues [18], although not discussed. FZD9 is a member of the frizzled family, characterized by multiple transmembrane domains, and thus, a predominantly membranous IHC signal would be expected. One possible explanation for the cytoplasmic localization of FZD9 is the clathrin‐dependent endocytic internalization mechanism proposed for other frizzled receptors such as FZD4 and FZD5, which has been shown to activate downstream signaling cascades [24, 25]. An additional interesting finding from the IHC analysis is the presence of FZD9 staining in blood vessels, although it was not possible to determine whether these represent neovasculature or pre‐existing vasculature. Further investigation is needed using full tumor and non‐tumor tissue sections. Moreover, Genotype‐Tissue Expression (GTEx) data show that normal skeletal muscle tissue exhibits high levels of FZD9. Given its role in intracellular calcium regulation, we speculate a potential involvement in this context. While blood vessels contain smooth muscle not covered in GTEx, they do possess calcium‐dependent mechanisms mediated by calmodulin, which is activated by calcium and may represent an important regulatory pathway for this ion [26].

FZD9 is a transmembrane protein that functions as a receptor for Wnt ligands. Different ligands have been investigated, leading to either pro‐tumorigenic or anti‐tumorigenic signaling outcomes. In 293 T cells, WNT2 acts as a ligand for FZD9, initiating signaling cascades that culminate in β‐catenin accumulation and increased cell proliferation rates [27]. In neuronal cells, WNT5 functions as a secreted growth factor, regulating dendritic spine formation in the rodent hippocampus. FZD9 forms a preassembled complex with the Gαo subunit of heterotrimeric G proteins, which dissociates upon WNT5A binding. Another mediator, the Gβγ subunits of heterotrimeric G proteins, is required to increase cytoplasmic calcium levels and promote spinogenesis, a process also mediated by WNT5A [28]. In non‐small cell lung cancer, Wnt7a binds to FZD9 and mediates antitumor effects that appear to be linked to G protein signaling. This interaction promotes the activation of an alternative pathway involving peroxisome proliferator‐activated receptor gamma (PPARG) and NF‐kappaB regulation [29]. In breast cancer, low levels of WNT5A or WNT7A have been described as predictors of poor survival [30, 31].

Returning to the role of FZD9 in breast cancer, the study conducted by Hachim et al. assessed protein expression by immunohistochemistry in 80 tumor samples, revealing a significant association between FZD9 levels and molecular subtype, with notably higher expression in triple‐negative tumors [18]. In contrast, in our patient cohort, we observed higher FZD9 expression among HER2‐positive cases, followed by TNBC and Luminal B tumors, suggesting potential biological variability between populations or methodological differences in clinical stratification. Additionally, transcriptomic data previously generated by our group also indicated increased FZD9 expression in basal‐like/TNBC tumors, raising the hypothesis that post‐transcriptional mechanisms and contextual factors, such as tumor microenvironment or mutational burden, may differentially modulate protein levels, conferring signaling plasticity to the Wnt/FZD9 axis in distinct molecular contexts of breast cancer.

We also investigated the epigenetic regulation of FZD9 in the HS‐578T cell line, which exhibited the lowest baseline expression levels among the models tested. Counterintuitively, exposure to the epigenetic agents Trichostatin‐A, a histone deacetylase inhibitor, and 5‐aza‐2′‐deoxycytidine, a DNA methyltransferase inhibitor, did not lead to transcriptional derepression of FZD9, as is commonly observed in genes silenced by promoter methylation. Instead, both treatments resulted in a further decrease in FZD9 expression levels. This finding suggests that FZD9 silencing in HS‐578T cells may not rely solely on classical epigenetic mechanisms, but may instead involve indirect transcriptional repression, compensatory inhibition by chromatin‐sensitive regulatory factors, or even negative feedback mechanisms triggered by global epigenetic interference. Given the pleiotropic and context‐dependent effects of global epigenetic modifiers, this observation should be interpreted as exploratory and does not, by itself, establish the mechanism of FZD9 regulation in HS‐578T cells. It may reflect indirect transcriptional effects, secondary chromatin remodeling events, or feedback regulatory circuits triggered by broad epigenetic interference, rather than a direct promoter demethylation‐driven response.

Both TSA and 5‐AZA have been investigated as therapeutic strategies in TNBC and non‐TNBC contexts, where distinct molecular mechanisms are involved. For instance, Wang et al. (2020) reported increased E‐cadherin and reduced vimentin expression in MCF7 cells following TSA treatment [32]. Song et al. (2018) performed experiments in TNBC cell lines and observed decreased levels of CYCLIN D1, CDK4, CDK6, and BCL‐XL, along with increased P21 expression after TSA exposure. When combined with doxorubicin, TSA showed a synergistic antiproliferative effect in HCC1806 and HCC38 cells [33]. The use of 5‐AZA has also been explored as a potential therapeutic agent in breast cancer subtypes. In HER2‐positive patients resistant to trastuzumab, 5‐AZA has shown promise in enhancing doxorubicin efficacy [34]. In the ER‐negative MDA‐MB‐231 cell line, 5‐AZA treatment restored estrogen receptor expression [35]. Moreover, pro‐apoptotic effects of cisplatin were amplified in TNBC lines following treatment with decitabine [36]. More specifically regarding FZD9, the study by Zhang et al. (2016) examined the effects of 5‐AZA in acute myeloid leukemia cells and concluded that FZD9 silencing was associated with promoter methylation and that its expression could be restored following demethylating treatment [37]. Together, these reports support that FZD9 can be epigenetically responsive in certain biological contexts, whereas our findings in breast cancer suggest a distinct and potentially indirect regulatory dynamic.

In response to the various cytotoxic agents tested, cisplatin, doxorubicin, paclitaxel, and ionizing radiation (6 Gy), we observed a markedly heterogeneous pattern of FZD9 expression modulation across the analyzed breast cancer cell lines. This variability was evident even within similar molecular subtypes, suggesting a regulatory mechanism dependent on both cell identity and the specific treatment administered. Although pronounced increases in FZD9 expression were detected in basal‐like models such as MDA‐MB‐231 following radiation exposure (fold change > 2) and paclitaxel, other cell lines with comparable profiles, including BT549 and HS‐578T, exhibited more modest or even inhibitory responses under the same conditions. Additionally, in luminal models such as MCF7, a robust suppression of FZD9 expression was observed, particularly after radiation, with fold change values below 0.2. These findings underscore the absence of a uniform regulatory pattern and indicate that FZD9 may exhibit distinct responses to genotoxic and chemotherapeutic stress depending on the biological context of the cell line. Notably, these observations are based on transcriptional readouts and should be interpreted as hypothesis‐generating until supported by protein‐level and functional validation.

The functional role of FZD9 in oncogenesis remains poorly defined and appears to be context‐dependent. In lung cancer, FZD9 has been associated with epithelial repair mechanisms and chemoprevention, with its silencing described as an early molecular event in tumorigenesis, particularly among smokers [38]. The use of prostacyclin analogs, such as iloprost, has been shown to restore FZD9 expression and prevent tumor progression in preclinical models, suggesting a tumor‐suppressive role in this setting. However, contrasting findings have emerged in other malignancies, including osteosarcomas and pancreatic tumors, where FZD9 may promote cell proliferation and survival [9, 12]. These opposing effects seem to be influenced by the availability of Wnt ligands and the type of intracellular signaling activated (canonical vs. non‐canonical), highlighting the functional complexity of this receptor in tumor biology.

This functional duality of FZD9 is likely influenced by the differential activation of Wnt ligands and the subsequent signaling pathways. Depending on the cellular context and ligand‐binding profile, FZD9 may activate either the canonical β‐catenin–dependent pathway [9] or non‐canonical routes [14, 29]. These pathways can exert opposing effects on tumor progression. In our study, the induction of FZD9 following chemotherapy and radiation in triple‐negative cell lines raises the possibility that this receptor participates in adaptive stress responses, potentially impacting cell survival or phenotypic plasticity. However, additional mechanistic studies are needed to determine whether this transcriptional modulation results in functionally relevant consequences for therapeutic resistance or tumor behavior.

This study has limitations, including its exploratory design and the relatively limited size of the study population, with small numbers of HER2‐positive and triple‐negative cases, which constrain definitive subtype‐specific comparisons and preclude robust multivariable survival analyses. The use of formalin‐fixed, paraffin‐embedded archival tissue, with variable storage times, may have influenced antigen preservation and immunoreactivity, potentially contributing to staining heterogeneity. In addition, the retrospective nature of the study relied on medical record data, which were incomplete for some clinicopathological variables and led to the exclusion of a subset of cases, thereby limiting sample size and clinical granularity. The absence of in vivo models or genetic manipulation experiments further restricts a direct assessment of FZD9 causality in tumor progression. Accordingly, the present findings should be interpreted as hypothesis‐generating rather than as evidence of a clinically validated biomarker. Future investigations should assess whether selective modulation of FZD9, either pharmacologically or via gene editing, can alter tumor phenotype or treatment sensitivity in specific breast cancer subtypes. The established use of iloprost as a FZD9‐restoring agent in lung cancer may serve as a rationale for exploring similar strategies in breast cancer, particularly in aggressive biological contexts, where FZD9 expression appears elevated.

## 5 Conclusion

Our findings demonstrate that FZD9 expression in breast cancer is heterogeneous and consistently associated with aggressive clinicopathological features, including HER2 positivity, high proliferative index, and reduced overall survival. In parallel, transcriptional analyses reveal pronounced context‐dependent modulation of FZD9 expression in response to cytotoxic and epigenetic therapies, underscoring the complexity of its regulation. Collectively, these results highlight the context‐specific role of FZD9 in breast cancer biology and support its consideration as an exploratory marker of tumor aggressiveness and therapy‐related biological responses, warranting further mechanistic investigation and validation in larger, independent study populations.

## Author Contributions

**Daniel Rodrigues de Bastos:** conceptualization, data curation, formal analysis, funding acquisition, investigation, methodology, project administration, supervision, writing – review and editing. **Ricardo Cesar Cintra:** data curation, formal analysis, writing—original draft. **Adhemar Longatto‐Filho:** formal analysis, writing—original draft. **Lara Termini:** supervision, writing—review and editing.

## Conflicts of Interest

The authors declare no conflicts of interest.

## Supporting information


**Supplementary Figure 1.** Immunohistochemical standardization of anti‐FZD9 staining in breast cancer tissue. **Supplementary Figure 2.** Immunohistochemical validation and antibody titration of anti‐FZD9 in glioblastoma samples. **Supplementary Figure 3.** Kaplan–Meier analysis of overall survival according to standard clinicopathological features in patients with invasive breast carcinoma.

## References

[1] F. Bray , M. Laversanne , H. Sung , et al., “Global Cancer Statistics 2022: Globocan Estimates of Incidence and Mortality Worldwide for 36 Cancers in 185 Countries,” CA: A Cancer Journal for Clinicians 74 (2024): 229–263, 10.3322/caac.21834. 38572751

[2] Estatísticas de câncer—Instituto Nacional de Câncer—INCA , accessed 27 Jun 2025, https://www.gov.br/inca/pt-br/assuntos/cancer/numeros.

[3] M. Luque‐Cabal , P. García‐Teijido , Y. Fernández‐Pérez , L. Sánchez‐Lorenzo , and I. Palacio‐Vázquez , “Mechanisms Behind the Resistance to Trastuzumab in HER2‐amplified Breast Cancer and Strategies to Overcome It,” supplement, Clinical Medicine Insights: Oncology 10, no. S1 (2016): 21–30, 10.4137/CMO.S34537. PMC4811269 27042153

[4] F. Carlino , C. Solinas , M. Orditura , M. D. Bisceglia , B. Pellegrino , and A. Diana , “Editorial: Heterogeneity in Breast Cancer: Clinical and Therapeutic Implications,” Frontiers in Oncology 14 (2024): 1321654, 10.3389/fonc.2024.1321654. 38469228 PMC10926019

[5] C. M. Perou , “Molecular Stratification of Triple‐Negative Breast Cancers,” Oncologist 16 (2011): 61–70, 10.1634/theoncologist.2011-S1-61. 21278442

[6] T. Sørlie , Y. Wang , C. Xiao , et al., “Distinct Molecular Mechanisms Underlying Clinically Relevant Subtypes of Breast Cancer: Gene Expression Analyses Across Three Different Platforms,” BMC Genomics 7 (2006): 127, 10.1186/1471-2164-7-127. 16729877 PMC1489944

[7] T. Sørlie , C. M. Perou , R. Tibshirani , et al., “Gene Expression Patterns of Breast Carcinomas Distinguish Tumor Subclasses With Clinical Implications,” Proceedings of the National Academy of Sciences 98 (2001): 10869–10874, 10.1073/pnas.191367098. PMC58566 11553815

[8] R. van Amerongen , A. N. Bowman , and R. Nusse , “Developmental Stage and Time Dictate the Fate of Wnt/β‐catenin‐ Responsive Stem Cells in the Mammary Gland,” Cell Stem Cell 11 (2012): 387–400, 10.1016/j.stem.2012.05.023. 22863533 PMC13155203

[9] Q. Wang , H. Liu , Q. Wang , et al., “Involvement of c‐Fos in Cell Proliferation, Migration, and Invasion in Osteosarcoma Cells Accompanied by Altered Expression of Wnt2 and Fzd9,” PLoS One 12 (2017): e0180558, 10.1371/journal.pone.0180558. 28665975 PMC5493424

[10] C. M. Zeng , Z. Chen , and L. Fu , “Frizzled Receptors as Potential Therapeutic Targets in Human Cancers,” International Journal of Molecular Sciences 19 (2018): 1543. 29789460 10.3390/ijms19051543 PMC5983605

[11] Y. K. Wang , C. H. Samos , R. Peoples , L. A. Perez‐Jurado , R. Nusse , and U. Francke , “A Novel Human Homologue of the Drosophila Frizzled Wnt Receptor Gene Binds Wingless Protein and Is in the Williams Syndrome Deletion at 7q11.23,” Human Molecular Genetics 6 (1997): 465–472, 10.1093/hmg/6.3.465. 9147651

[12] M. F. Zacarías‐Fluck , T. Jauset , S. Martínez‐Martín , et al., “The Wnt Signaling Receptor Fzd9 Is Essential for Myc‐Driven Tumorigenesis in Pancreatic Islets,” Life Science Alliance 4 (2021): e201900490, 10.26508/lsa.201900490. 33653688 PMC8008953

[13] Abdul , H. Rahman , N. F. M. Manzor , G. C. Tan , et al., “Upregulation of SOX‐2, FZD9, Nestin, OCT‐4 and FGF‐4 Expression in Human Chorion Derived‐Stem Cells After Angiogenic Induction,” supplement, Medical Journal of Malaysia 63, no. Suppl A (2008): 57–58. 19024982

[14] K. Sompel , L. D. Dwyer‐Nield , A. J. Smith , et al., “Iloprost Requires the Frizzled‐9 Receptor to Prevent Lung Cancer,” iScience 25 (2022): 104442, 10.1016/j.isci.2022.104442. 35707728 PMC9189122

[15] A. Heilmann , T. Schinke , R. Bindl , et al., “The Wnt Serpentine Receptor Frizzled‐9 Regulates New Bone Formation in Fracture Healing,” PLoS One 8 (2013): e84232, 10.1371/journal.pone.0084232. 24391920 PMC3877253

[16] Z. Zhang , J. Schittenhelm , K. Guo , et al., “Upregulation of Frizzled 9 in Astrocytomas,” Neuropathology and Applied Neurobiology 32 (2006): 615–624, 10.1111/j.1365-2990.2006.00770.x. 17083476

[17] M. A. Tennis , M. L. New , D. G. McArthur , D. T. Merrick , L. D. Dwyer‐Nield , and R. L. Keith , “Prostacyclin Reverses the Cigarette Smoke‐Induced Decrease in Pulmonary Frizzled 9 Expression Through miR‐31,” Scientific Reports 6 (2016): 28519, 10.1038/SREP28519. 27339092 PMC4919780

[18] M. Y. Hachim , I. Y. Hachim , I. M. Talaat , N. M. Yakout , and R. Hamoudi , “M1 Polarization Markers Are Upregulated in Basal‐Like Breast Cancer Molecular Subtype and Associated With Favorable Patient Outcome,” Frontiers in Immunology 11 (2020), 10.3389/fimmu.2020.560074. PMC7701279 33304345

[19] D. R. de Bastos , M. P. F. Conceição , A. P. P. Michelli , et al., “An in Silico Analysis Identified FZD9 as a Potential Prognostic Biomarker in Triple‐Negative Breast Cancer Patients,” European Journal of Breast Health 17 (2021): 42–52, 10.4274/ejbh.2020.5804. 33796830 PMC8006790

[20] A. Kassambara , Survminer: Drawing Survival Curves using 'ggplot2'. R package version 0.5.2, (2023), https://CRAN.R-project.org/package=survminer.

[21] T. Cokelaer , E. Chen , F. Iorio , et al., “GDSCTools for Mining Pharmacogenomic Interactions in Cancer,” Bioinformatics 34 (2018): 1226–1228, 10.1093/bioinformatics/btx744. 29186349 PMC6031019

[22] A. S. Linhares Moreira , T. M. Cunha , and S. Esteves , “Cervical Cancer Recurrence—Can We Predict the Type of Recurrence?,” Diagnostic and Interventional Radiology 26 (2020): 403–410, 10.5152/dir.2020.19437. 32815522 PMC7490029

[23] J. Jiménez‐Sánchez , J. J. Bosque , G. A. Jiménez Londoño , et al., “Evolutionary Dynamics at the Tumor Edge Reveal Metabolic Imaging Biomarkers,” Proceedings of the National Academy of Sciences 118 (2021): e2018110118, 10.1073/pnas.2018110118. PMC8017959 33536339

[24] H. Yamamoto , H. Komekado , and A. Kikuchi , “Caveolin Is Necessary for Wnt‐3a‐Dependent Internalization of LRP6 and Accumulation of β‐Catenin,” Developmental Cell 11 (2006): 213–223, 10.1016/j.devcel.2006.07.003. 16890161

[25] A. Yu , J.‐F. Rual , K. Tamai , et al., “Association of Dishevelled With the Clathrin AP‐2 Adaptor Is Required for Frizzled Endocytosis and Planar Cell Polarity Signaling,” Developmental Cell 12 (2007): 129–141, 10.1016/j.devcel.2006.10.015. 17199046 PMC2831292

[26] P. Pascual‐Vargas and P. C. Salinas , “A Role for Frizzled and Their Post‐Translational Modifications in the Mammalian Central Nervous System,” Frontiers in Cell and Developmental Biology 9 (2021): 692888, 10.3389/fcell.2021.692888. 34414184 PMC8369345

[27] T. Karasawa , H. Yokokura , J. Kitajewski , and P. J. Lombroso , “Frizzled‐9 Is Activated by Wnt‐2 and Functions in Wnt/β‐Catenin Signaling,” Journal of Biological Chemistry 277 (2002): 37479–37486, 10.1074/jbc.M205658200. 12138115

[28] V. T. Ramírez , E. Ramos‐Fernández , J. P. Henríquez , A. Lorenzo , and N. C. Inestrosa , “Wnt‐5a/Frizzled9 Receptor Signaling Through the Gαo‐Gβγ Complex Regulates Dendritic Spine Formation,” Journal of Biological Chemistry 291 (2016): 19092–19107, 10.1074/jbc.M116.722132. 27402827 PMC5009279

[29] R. A. Winn , M. Van Scoyk , M. Hammond , et al., “Antitumorigenic Effect of Wnt 7a and Fzd 9 in Non‐Small Cell Lung Cancer Cells Is Mediated Through ERK‐5‐dependent Activation of Peroxisome Proliferator‐Activated Receptor γ,” Journal of Biological Chemistry 281 (2006): 26943–26950, 10.1074/jbc.M604145200. 16835228

[30] K. Yi , K.‐W. Min , Y. C. Wi , et al., “Wnt7a Deficiency Could Predict Worse Disease‐Free and Overall Survival in Estrogen Receptor‐Positive Breast Cancer,” Journal of Breast Cancer 20 (2017): 361, 10.4048/jbc.2017.20.4.361. 29285041 PMC5743996

[31] Z. Zhong , M. Shan , J. Wang , T. Liu , Q. Shi , and D. Pang , “Decreased Wnt5a Expression Is a Poor Prognostic Factor in Triple‐Negative Breast Cancer,” Medical Science Monitor 22 (2016): 1–7, 10.12659/MSM.894821. 26721633 PMC4700863

[32] X. Wang , S. Chen , T. Shen , et al., “Trichostatin A Reverses Epithelial‐Mesenchymal Transition and Attenuates Invasion and Migration in MCF‐7 Breast Cancer Cells,” Experimental and Therapeutic Medicine 19, no. 3 (2020): 1687–1694, 10.3892/etm.2020.8422. PMC7027139 32104221

[33] X. Song , J. Q. Wu , X. F. Yu , X. S. Yang , and Y. Yang , “Trichostatin A Inhibits Proliferation of Triple Negative Breast Cancer Cells by Inducing Cell Cycle Arrest and Apoptosis,” Neoplasma 65 (2018): 898–906, 10.4149/neo_2018_181212N476. 30334455

[34] V. Buocikova , E. M. Longhin , E. Pilalis , et al., “Decitabine Potentiates Efficacy of Doxorubicin in a Preclinical Trastuzumab‐Resistant HER2‐positive Breast Cancer Models,” Biomedicine & Pharmacotherapy = Biomedecine & Pharmacotherapie 147 (2022): 112662, 10.1016/j.biopha.2022.112662. 35091237

[35] A. Salahuddin , H. Ghanem , G. A. Omran , and M. W. Helmy , “Epigenetic Restoration and Activation of ERβ: An Inspiring Approach for Treatment of Triple‐Negative Breast Cancer,” Medical Oncology 39 (2022): 150, 10.1007/s12032-022-01765-1. 35843988 PMC9288957

[36] W. Nakajima , K. Miyazaki , M. Sakaguchi , et al., “Epigenetic Priming With Decitabine Augments the Therapeutic Effect of Cisplatin on Triple‐Negative Breast Cancer Cells Through Induction of Proapoptotic Factor NOXA,” Cancers 14 (2022): 248, 10.3390/cancers14010248. 35008411 PMC8749981

[37] Y. Zhang , Q. Jiang , X. Kong , et al., “Methylation Status of the Promoter Region of the Human Frizzled 9 Gene in Acute Myeloid Leukemia,” Molecular Medicine Reports 14 (2016): 1339–1344, 10.3892/mmr.2016.5387. 27314612

[38] A. J. Smith , P. Do , K. Sompel , A. Elango , and M. A. Tennis , “miR‐520a‐5p Regulates Frizzled 9 Expression and Mediates Effects of Cigarette Smoke and Iloprost Chemoprevention,” Scientific Reports 12 (2022): 2388, 10.1038/s41598-022-06292-7. 35149732 PMC8837775

