# Supplementary material for: Frizzled‐9 Expression Is Associated With Aggressive Clinicopathological Features and Reduced Overall Survival in Invasive Breast Carcinoma
Source: Pathol Int. 2026 Mar 10;76(3):e70104. doi: 10.1111/pin.70104 (PMC12976460; doi:10.1111/pin.70104)
Supplement: Supplementary file 1 — Supplementary Figure 1. Immunohistochemical standardization of anti‐FZD9 staining in breast cancer tissue. Supplementary Figure 2. Immunohistochemical validation and antibody titration of anti‐FZD9 in glioblastoma samples. Supplementary Figure 3. Kaplan–Meier analysis of overall survival according to standard clinicopathological features in patients with invasive breast carcinoma. [file PIN-76-0-s001.docx]

**Supplementary material**


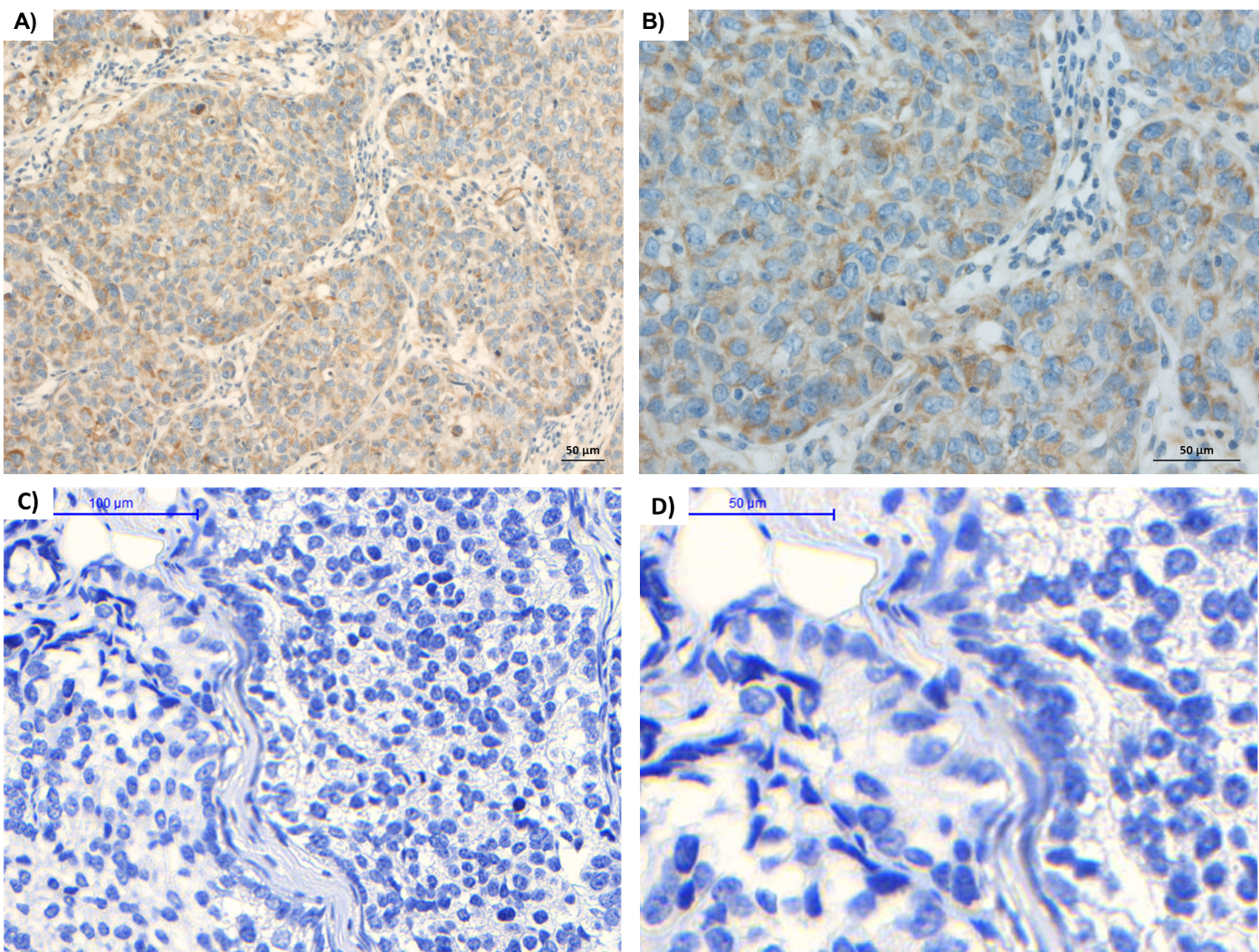


**Supplementary Figure 1.** Immunohistochemical standardization of anti-FZD9 staining in breast cancer tissue. Representative images from a triple-negative breast cancer specimen stained with anti-FZD9 antibody at a dilution of 1:500, showing moderate cytoplasmic immunoreactivity at 20× (A) and 40× (B) magnification. Negative control sections, derived from breast tumor tissue processed in parallel without primary antibody, are shown at 20× (C) and 40× (D) magnification, demonstrating the absence of nonspecific background staining. All assays were performed under identical experimental conditions to ensure staining specificity and reproducibility.


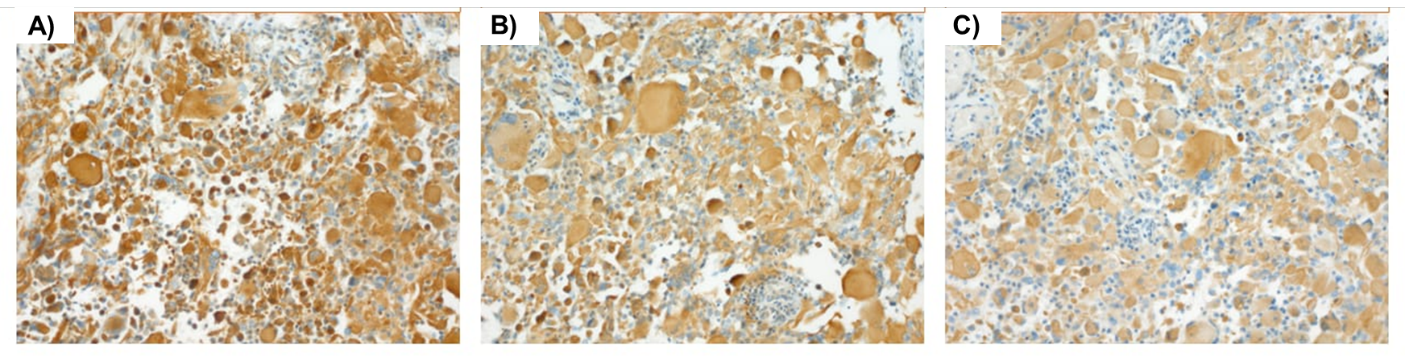


**Supplementary Figure 2.** Immunohistochemical validation and antibody titration of anti-FZD9 in glioblastoma samples. Representative immunohistochemical staining of FZD9 in glioblastoma biopsy specimens using increasing antibody dilutions: 1:250 (A), 1:500 (B), and 1:1000 (C). Images were acquired at 20× magnification. Experiments were performed in samples from different patients, all processed under identical conditions. For each staining run, negative control sections lacking primary antibody were included to assess background signal and confirm staining specificity. The images shown are representative of multiple independent assays.


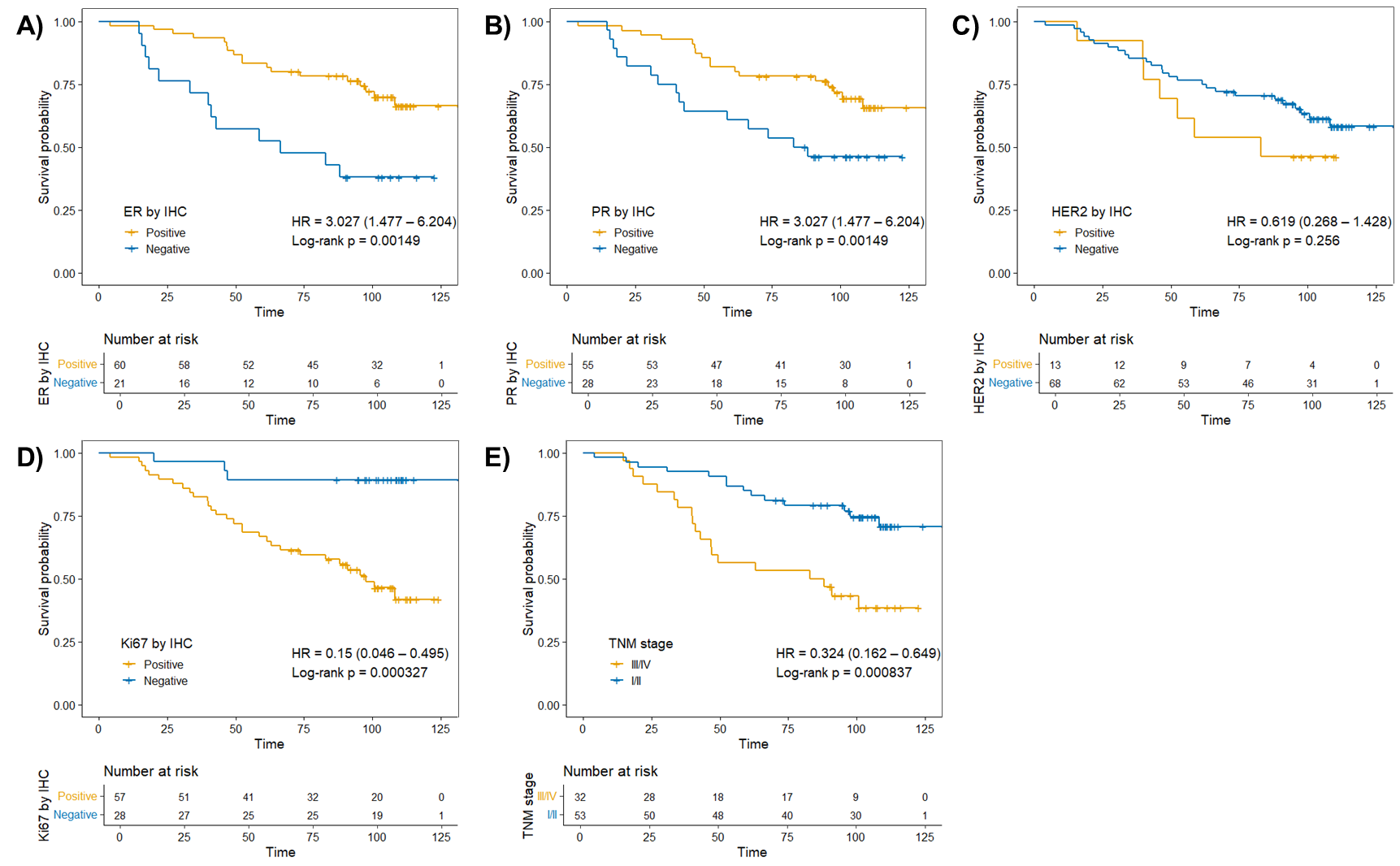


**Supplementary Figure 3.** Kaplan–Meier analysis of overall survival according to standard clinicopathological features in patients with invasive breast carcinoma. (A) Estrogen receptor (ER) status by IHC; (B) Progesterone receptor (PR) status by IHC; (C) HER2 status by IHC; (D) Ki-67 status (cut-off: 20%); (E) TNM clinical stage (grouped as I/II vs. III/IV).
